# Supplementary material for: Integrated care pathways in neurosurgery: A systematic review
Source: PLoS One. 2021 Aug 2;16(8):e0255628. doi: 10.1371/journal.pone.0255628 (PMC8328336; doi:10.1371/journal.pone.0255628)
Supplement: S1 Table — (DOCX) [file pone.0255628.s002.docx]

**S1 Table. Full search phrases used for the three respective databases.**

| **Ovid MEDLINE** | | | 493  articles |
| --- | --- | --- | --- |
| ICP concept | | | |
| 1 | exp Critical Pathways/ | | |
| 2 | Critical pathway*.tw. | | |
| 3 | exp “Delivery of Health Care, Integrated”/ | | |
| 4 | Integrated care pathway*.tw. | | |
| 5 | (multidisciplinary or multi-disciplinary or interdisciplinary or inter-disciplinary or integrated).tw. | | |
| 6 | (pathway* or protocol* or plan* or map*).tw. | | |
| 7 | 5 and 6 | | |
| 8 | 1 or 2 or 3 or 4 or 7 | | |
| Neurosurgery concept | | | |
| 9 | Exp Neurosurgery/ | | |
| 10 | Neurosurg*.tw | | |
| 11 | 9 or 10 | | |
| Combined concepts | | | |
| 12 | 8 and 11 | | |
| **Embase** | | | 1766 articles |
| ICP concept | | | |
| 1 | exp clinical pathway/ | | |
| 2 | Clinical pathway*.tw. | | |
| 3 | Exp integrated health care system/ | | |
| 4 | Integrated care pathway*.tw. | | |
| 5 | (multidisciplinary or multi-disciplinary or interdisciplinary or inter-disciplinary or integrated).tw. | | |
| 6 | (pathway* or protocol* or plan* or map*).tw. | | |
| 7 | 5 and 6 | | |
| 8 | 1 or 2 or 3 or 4 or 7 | | |
| Neurosurgery concept | | | |
| 9 | Exp Neurosurgery/ | | |
| 10 | Neurosurg*.tw | | |
| 11 | 9 or 10 | | |
| Combined concepts | | | |
| 12 | 8 and 11 | | |
| **Cochrane Controlled Register of Trials CENTRAL** | | 26 articles | |
| ICP concept | | | |
| 1 | MeSH descriptor: [Critical Pathways] explode all trees | | |
| 2 | (Critical pathway*):ti,ab,kw | | |
| 3 | MeSH descriptor: [Delivery of Health Care, Integrated] explode all trees | | |
| 4 | (Integrated care pathway*):ti,ab,kw | | |
| 5 | (multidisciplinary or multi-disciplinary or interdisciplinary or inter-disciplinary or integrated):ti,ab,kw | | |
| 6 | (pathway* or protocol* or plan* or map*):ti,ab,kw. | | |
| 7 | #5 AND #6 | | |
| 8 | #1 OR #2 OR #3 OR #4 OR #7 | | |
| Neurosurgery concept | | | |
| 9 | MeSH descriptor: [Neurosurgery] explode all trees | | |
| 10 | (Neurosurg*):ti,ab,kw | | |
| 11 | #9 OR #10 | | |
| Combined concepts | | | |
| 12 | #8 AND #11 | | |
