# Supplementary material for: Integrated care pathways in neurosurgery: A systematic review
Source: PLoS One. 2021 Aug 2;16(8):e0255628. doi: 10.1371/journal.pone.0255628 (PMC8328336; doi:10.1371/journal.pone.0255628)
Supplement: S2 Table — (DOCX) [file pone.0255628.s003.docx]

**S2 Table. Inclusion and exclusion criteria used to select studies for the review**

| Inclusion criteria | Exclusion criteria |
| --- | --- |
| - Original studies assessing the effect of an integrated care pathway for patients with neurosurgical diseases | - Not written in English - No original research data e.g. narrative and systematic reviews, editorials, commentaries, opinion papers, letters, education papers, conference abstracts, protocols, reports, theses or book chapters - Conference abstract only, no full text available. - Non-human participants (e.g. murine, porcine studies) - Not exclusively about neurosurgical diseases (not on a cohort of patients with neurosurgical disease at all, or on a cohort of patients with neurosurgical disease within a heterogenous trauma/plastics population, where data could not be disaggregated) - On a cohort of patients with neurosurgical disease but no outcomes of the integrated care pathway reported, that meets our definition - Overlapping population data |
