# Supplementary material for: Integrated care pathways in neurosurgery: A systematic review
Source: PLoS One. 2021 Aug 2;16(8):e0255628. doi: 10.1371/journal.pone.0255628 (PMC8328336; doi:10.1371/journal.pone.0255628)
Supplement: S3 Table — (DOCX) [file pone.0255628.s004.docx]

**S3 Table. Joanna Briggs Institute quality assessment checklist for cohort studies**

|  |  | | **Question no.** | | | | | | | | | |  | |  | |
| --- | --- | --- | --- | --- | --- | --- | --- | --- | --- | --- | --- | --- | --- | --- | --- | --- |
| **Study** | **1** | **2** | | **3** | **4** | **5** | **6** | **7** | **8** | **9** | **10** | **11** | | **Overall** | |  |
| Adogwa 2018 | ✔ | ✔ | | ✔ |  |  | ✔ | ✔ | ✔ | ✔ | ✔ | ✔ | | ✔ | |  |
| Bapat 2017 | ✔ | ✔ | | ✔ |  |  | ✔ | ✔ | ✔ | ✔ | ✔ | ✔ | | ✔ | |  |
| Bohl 2017 | ✔ | ✔ | | ✔ |  |  | ✔ | ✔ | ✔ | ✔ | ✔ | ✔ | | ✔ | |  |
| Brown 2018 | ✔ | ✔ | | ✔ |  |  | ✔ | ✔ | ✔ | ✔ | ✔ | ✔ | | ✔ | |  |
| Carminucci 2016 | ✔ | ✔ | | ✔ |  |  | ✔ | ✔ | ✔ | ✔ | ✔ | ✔ | | ✔ | |  |
| Chern 2010 | ✔ | ✔ | | ✔ |  |  | ✔ | ✔ | ✔ | ✔ | ✔ | ✔ | | ✔ | |  |
| Chung 2005 | ✔ | ✔ | | ✔ |  |  | ✔ | ✔ | ✔ | ✔ | ✔ | ✔ | | ✔ | |  |
| Jin 2008 | ✔ | ✔ | | ✔ |  |  | ✔ | ✔ | ✔ | ✔ | ✔ | ✔ | | ✔ | |  |
| Kurlander 2020 | ✔ | ✔ | | ✔ |  |  | ✔ | ✔ | ✔ | ✔ | ✔ | ✔ | | ✔ | |  |
| Namiranian 2018 | ✔ | ✔ | | ✔ |  |  | ✔ | ✔ | ✔ | ✔ | ✔ | ✔ | | ✔ | |  |
| Pritchard 2004 | ✔ | ✔ | | ✔ |  |  | ✔ | ✔ | ✔ | ✔ | ✔ | ✔ | | ✔ | |  |
| Sethi 2017 | ✔ | ✔ | | ✔ |  |  | ✔ | ✔ | ✔ | ✔ | ✔ | ✔ | | ✔ | |  |
| Wang 2020 | ✔ | ✔ | | ✔ |  |  | ✔ | ✔ | ✔ | ✔ | ✔ | ✔ | | ✔ | |  |

1. Were the two groups similar and recruited from the same population?

2. Were the exposures measured similarly to assign people to both exposed and unexposed groups?

3. Was the exposure measured in a valid and reliable way?

4. Were confounding factors identified?

5. Were strategies to deal with confounding factors stated?

6. Were the groups/participants free of the outcome at the start of the study (or at the moment of exposure)?

7. Were the outcomes measured in a valid and reliable way?

8. Was the follow up time reported and sufficient to be long enough for outcomes to occur?

9. Was follow up complete, and if not, were the reasons to loss to follow up described and explored

10. Were strategies to address incomplete follow up utilized?

11. Was appropriate statistical analysis used?
