# Supplementary material for: Integrated care pathways in neurosurgery: A systematic review
Source: PLoS One. 2021 Aug 2;16(8):e0255628. doi: 10.1371/journal.pone.0255628 (PMC8328336; doi:10.1371/journal.pone.0255628)
Supplement: Attachment — Submitted filename: Response to Reviewers.docx [file pone.0255628.s006.docx]

Professor Ehab Farag, MD FRCA FASA

Academic Editor

PLOS ONE

1 July 2021

Dear Professor Ehab Farag,

**Re. Integrated care pathways in Neurosurgery: A systematic review [PONE-D-21-08319]**

We thank you for the editorial and reviewers’ comments, and for providing us the opportunity to revise our manuscript. We appreciate the careful review and constructive suggestions from all reviewers and have responded to the comments below.

Following this letter are the review comments with our response in italics, including how and where the text was modified. Enclosed are two copies of the edited manuscript, one with the changes highlighted using the Microsoft Word Tracker system and a second copy with the changes in normal typeface. The revision has been developed in consultation with all co-authors, and each author has given approval to the final form of this revision.

Thank you for your consideration.

Keng Siang Lee and Benjamin Davies

Bristol Medical School, University of Bristol, Bristol, UK. Department of Clinical Neurosurgery, University of Cambridge, Cambridge, UK

Email: [kl17746@bristol.ac.uk](mailto:kl17746@bristol.ac.uk) (KSL)

Email: [bd375@cam.ac.uk](mailto:bd375@cam.ac.uk) (BD)

**Journal Requirements:**

When submitting your revision, we need you to address these additional requirements.

1. Please ensure that your manuscript meets PLOS ONE's style requirements, including those for file naming. The PLOS ONE style templates can be found at

<https://journals.plos.org/plosone/s/file?id=wjVg/PLOSOne_formatting_sample_main_body.pdf> and

[https://journals.plos.org/plosone/s/file?id=ba62/PLOSOne_formatting_sample_title_authors_affiliations.pdf](about:blank)

2. Please include captions for your Supporting Information files at the end of your manuscript, and update any in-text citations to match accordingly. Please see our Supporting Information guidelines for more information: <http://journals.plos.org/plosone/s/supporting-information>.

***Response:***

*Thank you for pointing this out. We have now formatted the revised manuscript accordingly to the journal’s requirement.*

**Comments to the Author**

1. Is the manuscript technically sound, and do the data support the conclusions?  The manuscript must describe a technically sound piece of scientific research with data that supports the conclusions. Experiments must have been conducted rigorously, with appropriate controls, replication, and sample sizes. The conclusions must be drawn appropriately based on the data presented.

Reviewer #1: Partly

***Response:***

*Thank you. We have responded further to your additional comments below.*

2. Has the statistical analysis been performed appropriately and rigorously?

Reviewer #1: I Don't Know

***Response:***

*Thank you. Given the heterogeneity in data reporting, formal meta-analysis of the included studies was not possible. Therefore, we felt it was more appropriate to report the data narratively, with descriptive statistics only. This has, we believe, enabled a comprehensive overview of studies developing ICPs for Neurosurgery, including a recognition of potential limitations that should inform their development in the future.*

3. Have the authors made all data underlying the findings in their manuscript fully available? The [PLOS Data policy](http://www.plosone.org/static/policies.action#sharing) requires authors to make all data underlying the findings described in their manuscript fully available without restriction, with rare exception (please refer to the Data Availability Statement in the manuscript PDF file). The data should be provided as part of the manuscript or its supporting information, or deposited to a public repository. For example, in addition to summary statistics, the data points behind means, medians and variance measures should be available. If there are restrictions on publicly sharing data—e.g. participant privacy or use of data from a third party—those must be specified.

Reviewer #1: Yes

***Response:***

*Thank you.*

4. Is the manuscript presented in an intelligible fashion and written in standard English? PLOS ONE does not copyedit accepted manuscripts, so the language in submitted articles must be clear, correct, and unambiguous. Any typographical or grammatical errors should be corrected at revision, so please note any specific errors here.

Reviewer #1: Yes

***Response:***

*Thank you.*

5. Review Comments to the Author  Please use the space provided to explain your answers to the questions above. You may also include additional comments for the author, including concerns about dual publication, research ethics, or publication ethics. (Please upload your review as an attachment if it exceeds 20,000 characters)

Reviewer #1: The manuscript does not discuss what it was meant to find out possibly due to lack of data. The discussion is also vague and extensive. Clearcut the goals and outcome with a focussed discussion would be better.

***Response:*** *Thank you – despite a comprehensive search strategy, there is somewhat limited published examples of ICP in surgery*

*Whilst we think these findings support value in their role, across different neurosurgical disciplines, our detailed analysis of their development, design and evaluation identified weaknesses compared to best practice in ICP development.*

*Due to the heterogeneity in the data, only a narrative synthesis was possible which has created a lengthy article. However, overall we feel that this is a comprehensive representation of the field currently, and therefore a useful resource for others to build on.*

*We have made various amendments to the text to highlight the aims, and clarify the message. These changes have been made particularly in the subsection ‘Future neurosurgical ICPs should consider the growing methodological literature around their design and evaluation’. We hope these now meet your approval.*

6. PLOS authors have the option to publish the peer review history of their article ([what does this mean?](https://journals.plos.org/plosone/s/editorial-and-peer-review-process#loc-peer-review-history)). If published, this will include your full peer review and any attached files.   If you choose “no”, your identity will remain anonymous but your review may still be made public. **Do you want your identity to be public for this peer review?** For information about this choice, including consent withdrawal, please see our [Privacy Policy](https://www.plos.org/privacy-policy).

Reviewer #1: No

***Response:***

*Thank you for taking time to review our manuscript and providing constructive feedback which could only improve our work.*
